# Molecular dynamics simulations suggest the potential toxicity of fluorinated graphene to HP35 protein via unfolding the α-helix structure

**DOI:** 10.1038/s41598-024-59780-3

**Published:** 2024-04-22

**Authors:** Fangrong Zou, Zonglin Gu, Jose Manuel Perez-Aguilar, Yuqi Luo

**Affiliations:** 1 Department of Gastrointestinal and Hepatobiliary Surgery, Shenzhen Longhua District Central Hospital, No. 187, Guanlan Road, Longhua District, Shenzhen, 518110 Guangdong Province China; 2 College of Physical Science and Technology, Yangzhou University, Jiangsu, 225009 China; 3 School of Chemical Sciences, Meritorious Autonomous University of Puebla (BUAP), 72570 University City, Puebla, Mexico

**Keywords:** Fluorinated graphene, HP35 protein, Unfolding, Toxicity, Molecular dynamics simulation, Nanotoxicology, Nanoscale biophysics

## Abstract

Fluorinated graphene, a two-dimensional nanomaterial composed of three atomic layers, a central carbon layer sandwiched between two layers of fluorine atoms, has attracted considerable attention across various fields, particularly for its potential use in biomedical applications. Nonetheless, scant effort has been devoted to assessing the potential toxicological implications of this nanomaterial. In this study, we scrutinize the potential impact of fluorinated graphene on a protein model, HP35 by utilizing extensive molecular dynamics (MD) simulation methods. Our MD results elucidate that upon adsorption to the nanomaterial, HP35 undergoes a denaturation process initiated by the unraveling of the second helix of the protein and the loss of the proteins hydrophobic core. In detail, substantial alterations in various structural features of HP35 ensue, including alterations in hydrogen bonding, Q value, and RMSD. Subsequent analyses underscore that hydrophobic and van der Waals interactions (predominant), alongside electrostatic energy (subordinate), exert influence over the adsorption of HP35 on the fluorinated graphene surface. Mechanistic scrutiny attests that the unrestrained lateral mobility of HP35 on the fluorinated graphene nanomaterial primarily causes the exposure of HP35's hydrophobic core, resulting in the eventual structural denaturation of HP35. A trend in the features of 2D nanostructures is proposed that may facilitate the denaturation process. Our findings not only substantiate the potential toxicity of fluorinated graphene but also unveil the underlying molecular mechanism, which thereby holds significance for the prospective utilization of such nanomaterials in the field of biomedicine.

## Introduction

Carbon-based nanomaterials (CBNs) have emerged as a focal point of research in recent decades since the seminal discoveries of fullerene C60 in 1985, carbon nanotubes (CNTs) in 1991, and graphene in 2004^1–3^. Due to their distinctive and remarkable attributes, including a high specific surface area, size-related and dimensional effects, extensive structural adaptability, as well as exemplary mechanical, electrical, and optical properties, CBNs have garnered substantial attention from diverse scientific fields^4–7^, and found applications as gas storage devices, transistors, sensors, nanocarriers, nanodrugs, and more^8–13^. As an illustration of the CBN’s applicability, the graphene nanosheet, an illustrious member of CBNs, has demonstrated remarkable antibacterial activity, rendering it suitable for application as an antibacterial agent^14–16^. Along these lines, the computational technique of molecular dynamics simulations aided to unveil two underlying antibacterial mechanisms: (i) graphene insertion into membranes and (ii) graphene extraction of lipids, both events driven by dispersion interactions^17^. Graphene oxide, a derivative of pristine graphene that is modified through surface oxidation, served as an intelligent platform carrying ovalbumin and exhibiting exceptional efficacy as a cancer vaccine^18^. Post-vaccine injection, the immune system is activated which initiates an efficient anti-tumor T-cell response. Another CBNs, Gd@C_82_(OH)_22_, a variant of metal-fullerol, has been tested as a nanodrug and showed to possess anti-pancreatic adenocarcinoma activity by exerting direct inhibition on the matrix metalloproteinases 9 (MMP-9), which ultimately restraining the proliferation of cancer cells^19^. Although different CBNs exhibit versatile promising biomedical applications, the potential biosafety of these materials should be considered prior to the formal applications.

Fluorinated graphene, another member of the CBNs materials, is derived via the fluorination of both faces of the graphene surface. Numerous studies have showed the distinctive bandgap, optical properties, low surface energy, tribological characteristics, commendable thermal and chemical stability, and magnetic attributes inherent in the fluorinated graphene material^20–25^. According to these exceptional traits, fluorinated graphene finds diverse applications across various domains such as battery and electrochemistry, lubrication, self-cleaning, oil–water separation, thermally conductive yet electrically insulating materials, gas detection, storage, and separation, as well as in various biomedical applications^22,26–30^. Notably, researchers have identified significant promise in the biomedical field, owing to the intriguing C-F bonds that elicit biological and paramagnetic responses^31–34^. Loh et al.^35^ utilized fluorinated graphene as a scaffold for the growth of mesenchymal stem cell (MSC), revealing heightened cell adhesion, proliferation, and a neuro-inductive effect through spontaneous cell polarization. Also, Ajayan et al.^33^ highlighted the role of fluorinated graphene oxide (GO) as an exceptional carbon-based magnetic resonance imaging (MRI) contrast agent, free of magnetic nanoparticles. Their findings propose the potential development of fluorinated graphene as a theranostic material for multimodal imaging, including MRI, ultrasound, and photoacoustics. Despite extensive efforts devoted to exploring the promising applications of fluorinated graphene, the potential nanotoxicology of this nanomaterial, particularly at the molecular level, remains unclear. The extensive applications of any nanomaterial will increase their exposure to water, soil, air and living organisms such as plant, animals and even human beings; for instance, scientists have found anthropogenic carbon nanotubes in the airways of Parisian children^36^. HP35 is found in the brush border of the intestine and in part of the renal epithelium. The nanomaterial may enter into intestine and renal epithelium, by which the nanomaterial may interact with HP35. One way to evaluate the biocompatibility of nanostructures is to investigate their interaction with biomolecules to characterize the structural and functional consequences that such interaction elicit in the biomolecule. In this context, one of the model proteins utilized to investigate the interaction protein-nanomaterial is the 35-residue Villin headpiece subdomain (HP35). In addition to be one of the most characterized globular proteins, HP35 is a suitable choice to investigate the nanomaterial’s biocompatibility since it is found in the brush border of the intestine and in part of the renal epithelium, one of the possible way in which the nanomaterial could enter into the body and hence increasing a possible encounter between the nanomaterial and HP35. The HP35 protein is a small fast-folding protein that displays the general properties associated with common globular proteins and structurally, it consists of three α-helices. HP35 has undergone extensive experimental and computational investigation regarding its folding and unfolding dynamics that has been helpful to better understand the protein folding problem^37–39^. More importantly, owing to these characteristics, various theoretical studies have extensively utilized this protein model to assess the potential biocompatibility of various nanomaterials at a from molecular level through its direct interaction^40–44^, with graphene^45^, defective graphene^46^, graphene quantum dots^47^, boron nitride^48^, phosphorene^49^, carbon nitride (C_2_N^50^, C_3_N_4_^51^, C_3_N_3_^52^ and C_3_N^53^), carbon boride (BC_3_)^54^, and α-phase phosphorene carbide^55^, to mention a few. Researchers usually used the HP35 protein to investigate the possible protein unfolding on the nanomaterials’ surface, by which the potential bio-effect could be evaluated via observing the perturbations in the secondary and tertiary structure (i.e., unfolding) of this protein after adsorption onto the nanomaterials. Specifically, the nanomaterial is usually considered biocompatible if the HP35 protein could maintain its structural integrity upon adsorption onto the nanomaterial’s surface, whereas the nanomaterial is considered toxic if HP35 encountered structural unfolding after adhering onto nanostructure. Therefore, following the acknowledged protocol as illustrated above, we herein use the HP35 as a representative globular protein to unveil the potential bio-effect and the underlying molecular mechanism of the fluorinated graphene nanomaterial. In this investigation, we explore the adsorption of the protein model HP35 onto fluorinated graphene using an unbiased MD simulation approach. Our MD simulation results suggest that HP35 can undergo unfolding after adsorption onto fluorinated graphene. The primary forces driving the adsorption of HP35 on fluorinated graphene are the hydrophobic and van der Waals interactions, with electrostatic interactions playing a minor role. The unfolding mechanism of HP35 on fluorinated graphene is attributed to the unrestricted and rapid movement of HP35 on the fluorinated graphene surface.

## Method

**Figure 1:**
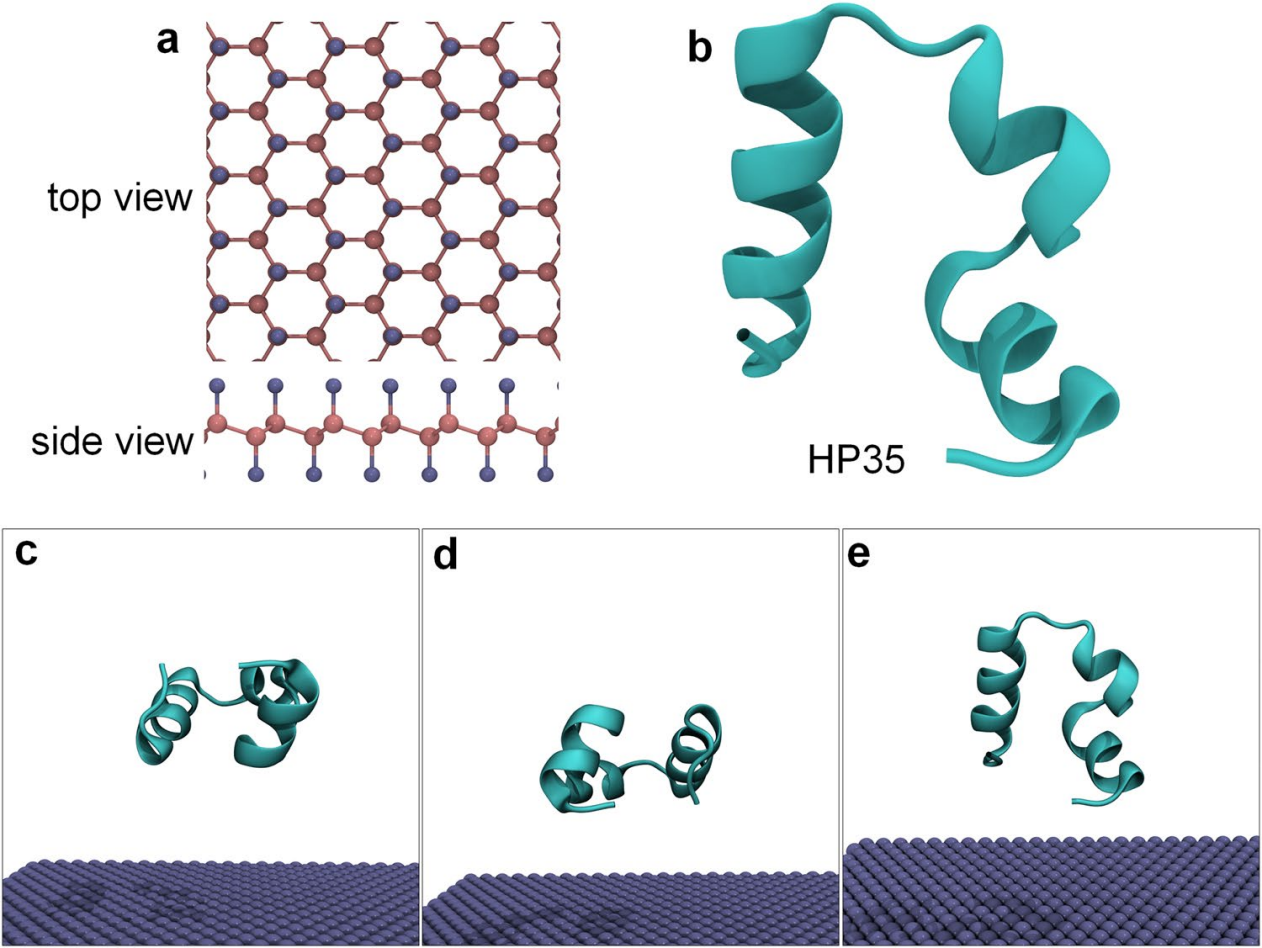
(**a**) Fluorinated graphene model. Fluorine and carbon atoms are showed by iceblue and pink spheres, respectively. (**b**) HP35 structure. (**c**,**d**) Initial configurations of sys-1 (**c**), sys-2 (**d**), and sys-3 (**e**).

Figure 1a and b showed the structures of the initial structures of the fluorinated graphene nanostructure and the HP35 protein. Three simulation systems were constructed, as depicted in Fig. 1c,d, where different orientation of the HP35, relative to the surface, are used (HP35 rotated at 0°, 180°, and 270°). These systems were denoted as sys-1, sys-2, and sys-3, respectively. In each system, the initial distance between the HP35 and the fluorinated graphene exceeded 1.2 nm (the separation distance was utilized based on previous studies regarding nano-bio interaction)^56,57^, wherein such initial distance exclude any interaction between the biomolecules and the nanomaterial. The dimensions of the fluorinated graphene nanosheet are 6.08 × 6.98 nm^2^ along the x and y directions, while the simulation system's size along the z direction was set to 6.00 nm. Each system was immersed in a NaCl solution with a 0.15 M concentration of NaCl.

Molecular dynamics (MD) simulations were executed using the GROMACS software package (version 2018)^58^, and the generated trajectories were analyzed employing the VMD software^59^. The CHARMM27 force field^60^ was used to describe the protein molecule interactions and the TIP3P water model^61^ was utilized to characterize the water molecules. The force field parameters for the fluorinated graphene were adopted from a previous study^62^. The protein HP35 model was download from RCSB Protein Data Bank (PDB ID: 1YRF)^63^. The temperature was upheld at 300 K via the v-rescale thermostat^64^, and the pressure was maintained at 1 atm using the Parrinello-Rahman barostat^65^ with semi-isotropic pressure coupling in the z direction (with no pressure applied in the x + y direction). Periodic boundary conditions were enforced in all dimensions. Long-range electrostatic interactions were treated utilizing the particle mesh Ewald (PME) method^66,67^, while van der Waals (vdW) interactions were computed within a cutoff distance of 1.2 nm. Bonds involving hydrogen atom were constrained to their equilibrium values using the LINCS algorithm^68^, while the SETTLE algorithm^69^ was employed to maintain the geometry of the water molecules. Throughout the simulations, the position of the fluorinated graphene nanostructure remained fixed. A time step of 2.0 fs was applied and the coordinates of all atoms were saved every 10 ps. Each system, namely sys-1, sys-2, and sys-3, was investigated by three parallel trajectories with each trajectory lasting 3500 ns.

## Results

Fluorinated graphene, a two-dimensional nanomaterial with three atomic layers, comprises a central carbon atom layer sandwiched by two layers of fluorine atoms. This configuration arises from the fluorination of graphene, as depicted in Fig. 1a. In contrast to the planar structure of pristine graphene, the carbon layer in fluorinated graphene, adopts an "armchair" configuration due to the sp^3^ hybridization of all carbon atoms. The two fluorine atom layers uniformly distribute across both basal faces. To explore the potential impact of HP35 upon adsorption onto fluorinated graphene, three initial setups were generated, introducing different orientations of the HP35 protein (rotated at 0°, 180°, and 270°); each system underwent three parallel simulations (run-1, run-2, and run-3).

**Figure 2:**
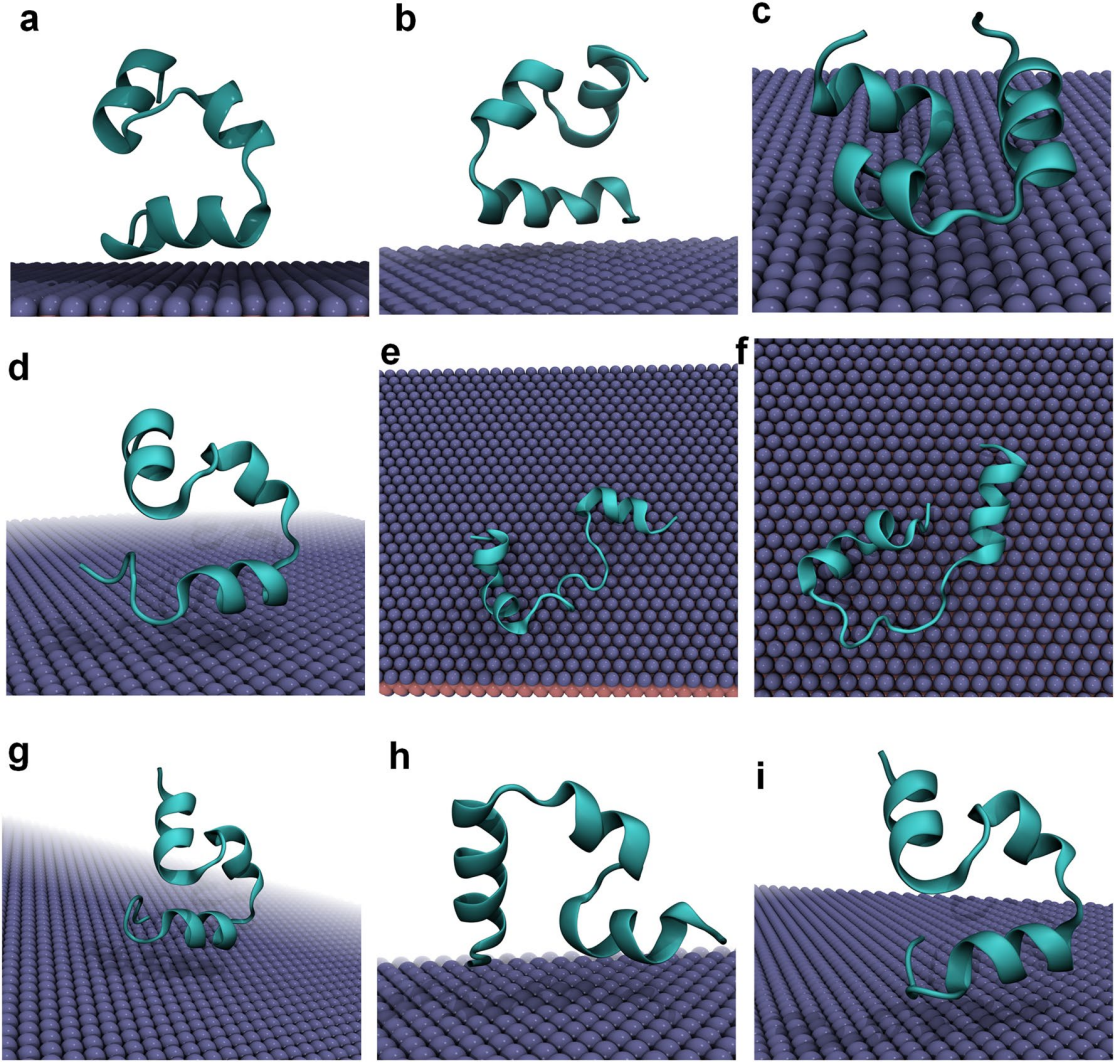
Final bound conformations. (**a**–**c**) The final bound conformations of three parallel simulations of sys-1. (**d**–**f**) The final bound conformations of three parallel simulations of sys-2. (**g**–**i**) The final bound conformations of three parallel simulations of sys-3.

To contrast with our fluorinated graphene simulations, we first performed an unbiased MD simulation of an individual HP35 protein in solution, as shown in Figure S1. Clearly, the entire conformation of the three α-helices of HP35, are retained along the 3500 ns simulation. In addition, the hydrogen bond and Q value calculations also show limited fluctuations, suggesting that the tertiary structure of HP35 remains stable in solution. In addition, a previous study also showed that HP35 maintains its 3D-structure, supporting our results^53^. Figure 2 illustrates the final configurations of HP35 bound to the surface of the fluorinated graphene in all trajectories. Strikingly, in certain trajectories, HP35 undergoes unfolding to different extents. Notably, run-2 and run-3 of sys-2 exhibit a pronounced denaturation in the second α-helix of HP35, whereas some trajectories reveal a milder unwinding at the protein's helical tail (e.g., run-2 of sys-1, run-1 of sys-2, and run-1 of sys-3, as shown in Figure S2). Given the potential loss of native structure of HP35 on the fluorinated graphene surface, a well-folded and well-behave globular protein, our results suggest that the fluorinated graphene material may exhibit a potential toxicity for globular proteins. Furthermore, in run-2 and run-3 of sys-2, the second helix of HP35 completely unravels and the entire protein nearly packs onto the fluorinated graphene surface, indicating a deleterious impact of this nanomaterial on HP35 structure (i.e., toxicity).

**Figure 3:**
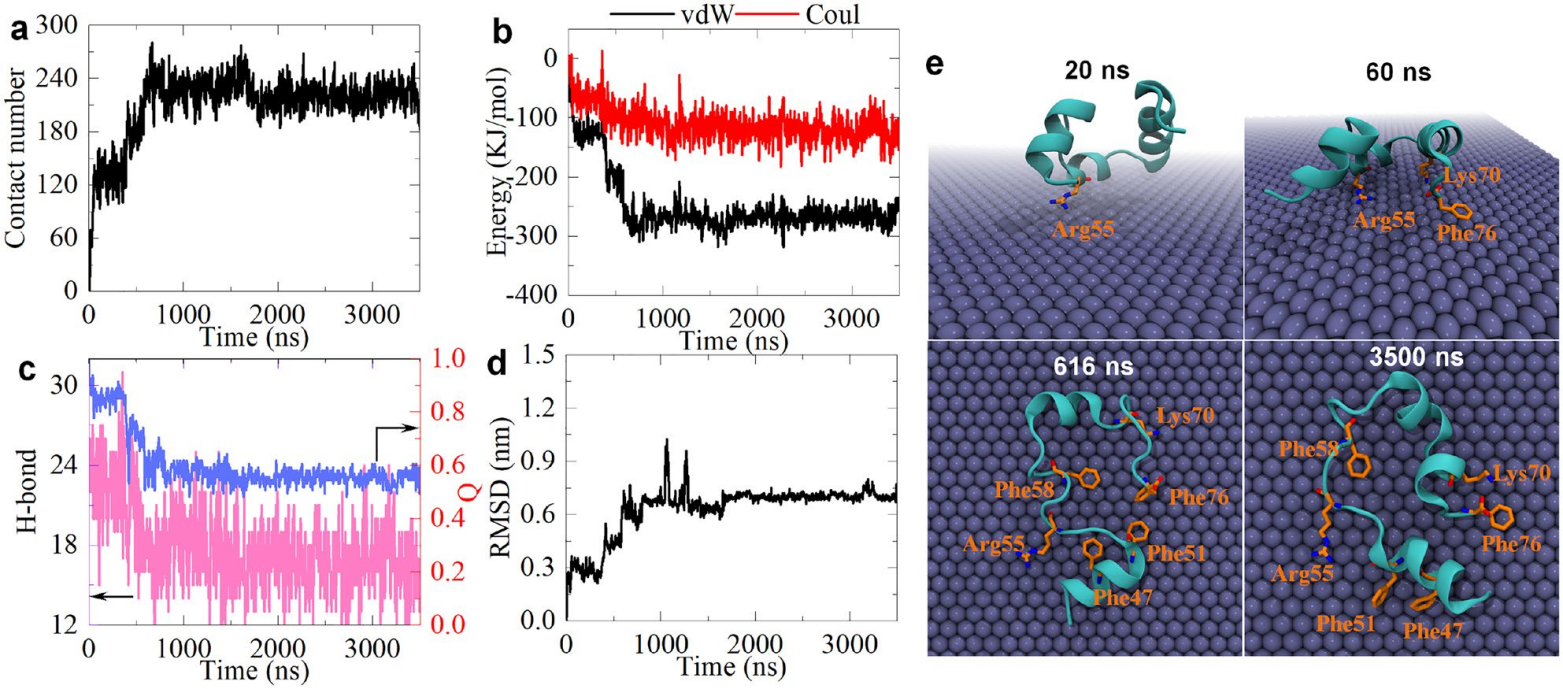
Analysis of the simulation run-3 of sys-2. (**a**) Atom contact number between HP35 and fluorinated graphene. (**b**) Interaction energies between HP35 and the fluorinated graphene. vdW and Coul indicate van der Waals and Coulomb energies, respectively. (**c**) Hydrogen bond number and Q value of HP35. (**d**) Root mean square deviation (RMSD) of HP35. (**e**) Snapshots of HP35 binding to fluorinated graphene at some time points. The contacted basic and aromatic residues are indicated.

To further elucidate the adsorption/unfolding dynamics of HP35 on the surface of fluorinated graphene, we selected a representative trajectory (run-3 of sys-2) for a comprehensive analysis, as depicted in Fig. 3. The detailed examination contains the contact number and interaction energy (both vdW and Coulomb energies) between the fluorinated graphene and HP35, the hydrogen bond number and Q value of HP35, root-mean-square deviation (RMSD) of HP35, and snapshots highlighting contacted basic and aromatic residues at critical time points. Initiating at 20 ns, HP35 establishes initial contact with fluorinated graphene via the basic residue Arg55. A residue contact is defined if any atom of the residue has a distance to the fluorinated graphene surface less than 0.5 nm. Based on the original contact of Arg55 and its corresponding high contact probability (see below), we conclude that Arg55 has a high preference to directly interact to the fluorinated graphene material. Next, we observed a sharply increase in the contact number value, to reach 67, with a corresponding increment in both vdW and Coulomb interaction energies, -72.8 and -28.7 kJ/mol, respectively. At this stage, the structural integrity of the HP35 structure remains robust, as evidenced by a high hydrogen bond number (24) and Q value (0.91), coupled with a low RMSD (0.27 nm). By 60 ns, the adhesion event intensifies, with one basal side of HP35 closely approaching the fluorinated graphene surface, leading to an increase contacts with residues Lys70 and Phe76. The contact number grows to 142, and the vdW and Coulomb energies rise to -123.3 and -54.6 kJ/mol, respectively. Hydrogen bond and Q values remain high (24 and 0.86), and the RMSD of HP35 shows a slight increase (approaching 0.37 nm). From 60 to 350 ns, the adsorption reaches a transient metastable state, with no obvious alterations in the aforementioned structural parameters indicating structural stability. Between 350 and 616 ns, a significant unfolding of HP35 occurs, that is, as depicted in the binding conformation shown in Fig. 3e, the second helix undergoes complete denaturation, resulting in a HP35 structure lying entirely on the surface of fluorinated graphene. Additional residues, including Phe58, Phe47, and Phe51, adhere to the fluorinated graphene surface. Consequently, the contact number and interaction energies suffer significant increments (contact number: 250; vdW energy: -266.3 kJ/mol; Coulomb energy: -123.0 kJ/mol). Simultaneously, hydrogen bond and Q values sharply decrease to 18 and 0.62, respectively, while the RMSD increases to 0.64 nm. Moreover, the analyses performed in the simulation run-2 of sys-2, also demonstrate similar results (Figure S3). We also analyze the secondary structure content of HP35 during its unfolding onto the fluorinated graphene, as shown in Figure S4. Clearly, the second α-helical structure of HP35 completely disappeared, supporting the above findings. Then, we evaluate the conformational entropy of HP35 as a function of time via the following formula: $S=-P{\text{ln}}P$, wherein *P* indicates the ratio of the residue numbers of α-helix of HP35 at each time point with respective to the one of HP35 original structure. As shown in Figure S5, after the HP35 unfolding on fluorinated graphene, the conformational entropy value of HP35 increased. Collectively, these observations demonstrate the severe damage in the HP35 structure during this period. From 616 to 3500 ns, the adsorption remains stable, and the HP35 structure does not exhibit any recovery. Throughout the adsorption process, aromatic and basic protein residues play pivotal roles in mediating the interaction and unfolding of HP35 on the fluorinated graphene. Notably, numerous aromatic and basic residues contribute to intimate interfacial contact, including Phe47, Phe51, Phe58, Phe76, Arg55, and Lys70. Specifically, aromatic residues adhere to the highly aromatic fluorinated graphene surface guided by hydrophobic and vdW interactions (fluorinated graphene has a water contact angle of larger than 70°^62^). This interaction is very similar to the observed aromatic residue-graphene interactions, given the abundant benzene rings in the fluorinated graphene nanomaterial, resulting in π–π stacking interactions with aromatic residues. On the other hand, basic residues are driven by electrostatic and vdW interactions that arises from the interaction of the positively charged basic residues and the negatively charged fluorine atoms exposed on both basal faces of fluorinated graphene nanomaterial.

Apart from the already described trajectories that displayed robust denaturation of HP35, we also analyzed in more detailed the other trajectories, as shown in Figure S6-S12. Clearly, compared with the results from run-2 and run-3 of sys-2, these trajectories show a more moderate interaction between HP35 and fluorinated graphene, reflecting by the lower contact number and interaction energies. In addition, the hydrogen bond number evolution, Q value, and RMSD in these trajectories clearly demonstrate that the structure of HP35 upon adsorption onto the fluorinated graphene presents mild alteration. We note that the trajectories where the HP35 structure suffered the larger local perturbations (i.e., run-2 of sys-1, run-1 of sys-2 and run-1 of sys-3 as shown in Figure S2) have an overall loss in its structural integrity. Specifically, in run-2 of sys-1, run-1 of sys-2 and run-1 of sys-3, the Q values finally decline to ~ 0.8, and meanwhile the corresponding RMSDs increase to over 0.3 nm. Furthermore, the key residues associated to the interfacial binding are also highlighted in each figure. Interestingly, the C-terminal residue Phe76, often participates in the interaction between HP35 and the fluorinated graphene, as observed in the trajectories of run-2 and run-3 of sys-2, suggesting the significance of this particular aromatic residue.

**Figure 4:**
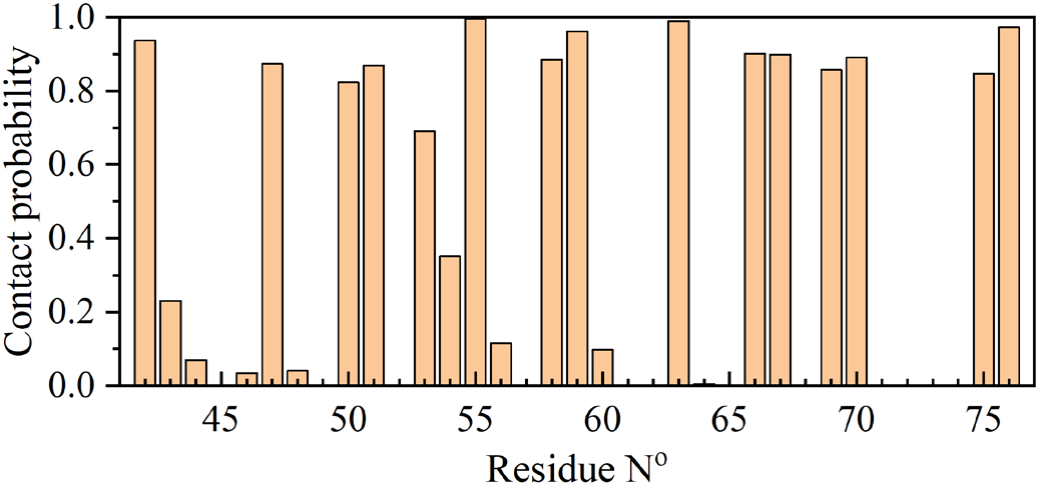
Contact probability of each residue to fluorinated graphene in the simulation run-3 of sys-2.

Considering the pivotal roles of aromatic and basic residues in guiding the adsorption of HP35 onto the fluorinated graphene surface, we summarize the contact probabilities of each residue bound to the nanomaterial, as illustrated in Fig. 4. Notably, certain residues exhibit exceptionally high contact probabilities, underscoring their significant contributions to the unfolding process of HP35. To identify these critical residues, we highlight those with contact probabilities surpassing 0.7, as presented in Table 1. Remarkably, all highlighted residues in Fig. 4 demonstrate elevated contact probabilities: 0.87 for Phe47, 0.87 for Phe51 (both located in the first helix), 0.99 for Arg55, 0.88 for Phe58 (both located in the second helix), 0.89 for Lys70 (located in the third helix), and 0.97 for Phe76 (C-terminal residue). This affirms the crucial roles of basic and aromatic residues in guiding the adsorption and unfolding of HP35 on the fluorinated graphene surface. Additionally, among the 14 residues with the highest contact probabilities, 10 residues (Leu42, Phe47, Val50, Phe51, Phe58, Ala59, Leu63, Leu69, Leu75, and Phe76) are hydrophobic residues (either aromatic or aliphatic residues), constituting 71.4% of the total high contacting residues. This suggests the critical role of hydrophobic interactions during the HP35-fluorinated graphene adsorption. In contrast, basic residues, represented by Arg55 and Lys70, account for only 14.3%, indicating a minor contribution from the electrostatic interactions. Consequently, the robust adsorption of the HP35 protein on the fluorinated graphene nanomaterial are predominantly driven by hydrophobic and van der Waals interactions, with electrostatic interactions playing a supplementary role, as further supported by Fig. 3b.

**Table 1:** The contact probabilities of some residues with their corresponding value larger than 0.7.

Residue name	Leu42	Phe47	Val50	Phe51	Arg55	Phe58	Ala59	Leu63	Gln66	Gln67	Leu69	Lys70	Leu75	Phe76
Contact probability	0.93	0.87	0.82	0.87	0.99	0.88	0.96	0.99	0.90	0.89	0.86	0.89	0.85	0.97

**Figure 5:**
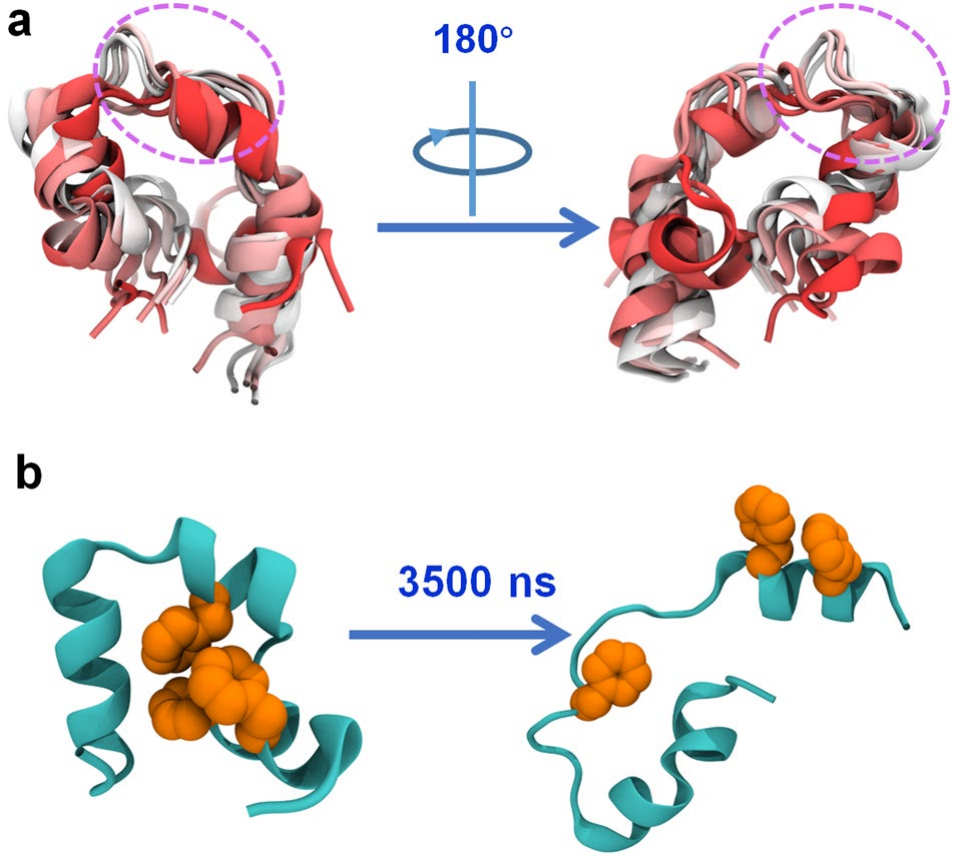
(**a**) Alignment of HP35 structures. The structures are extracted every 500 ns from the simulation run-3 of sys-2, yielding seven structures. The color evolution from red to white denote the HP35 at 0 ns to 3500 ns. (**b**) The hydrophobic core formed by three phenylalanines at 0 ns and 3500 ns. The HP35 and three phenylalanines, Phe47, Phe51, and Phe58, are showed by orange spheres.

We also align the HP35 structures at every 500 ns throughout the entire simulation, as depicted in Fig. 5a. Clearly, the second helical segment of HP35 is entirely absent after 1000 ns, and the unraveled structure persists until the conclusion of the 3500 ns simulation. Additionally, we observe that three aromatic residues (Phe47, Phe51, and Phe58) within the HP35 interior form a hydrophobic core that maintains the tertiary structure of HP35. To scrutinize the hydrophobic core in HP35, we plot the conformations of these three aromatic residues at the initial and final frames of the trajectory, as illustrated in Fig. 5b. Initially, the three phenylalanine residues intimately interact with each other, creating a hydrophobic core; however, as the simulations progress, the core is completely disrupted with one phenylalanine (Phe58, located in the second helix) positioned distant from the other two. Meanwhile, the three phenylalanine residues tightly bind to the fluorinated graphene surface (Fig. 3e). These findings further substantiate that HP35 undergoes substantial structural damage, including the break of the protein’s hydrophobic core, after adsorption onto fluorinated graphene nanosheet, supporting the potential toxicity of the fluorinated graphene to HP35.

**Figure 6:**
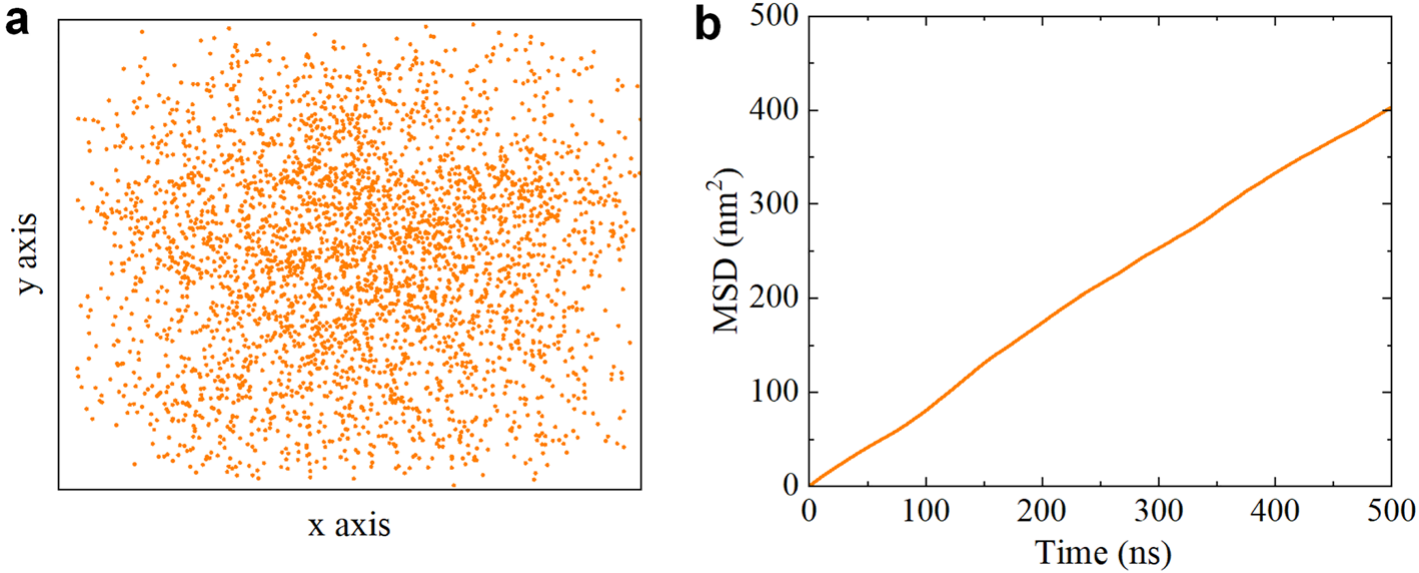
(**a**) Center of mass (CoM) positions of HP35 projecting onto x–y plane in the simulation run-3 of sys-2. Orange dots denote the x–y positions throughout the trajectory. (**b**) Mean square deviation (MSD) of HP35 moving along x–y plane.

Finally, to elucidate the mechanism underlying the unfolding of HP35 on fluorinated graphene, we conduct additional analyses, including the position of HP35 projected onto the fluorinated graphene surface and the mean square displacement (MSD) of HP35 (Fig. 6). Remarkably, HP35 exhibits unrestricted and rapid movement on fluorinated graphene, resulting in a uniform distribution of HP35 positions and a high MSD value. In line with prior research, the unconstrained and fast movement of HP35 on 2D nanomaterials (e.g., C_3_N)^53^ can potentially expose aromatic residues in the hydrophobic core, ultimately triggering the unfolding of HP35. Consequently, the unfolding of HP35 on fluorinated graphene is predominantly ascribed to the free and rapid movement of HP35 on the fluorinated graphene surface as well as the strong interactions with aromatic residues.

Considering that many previous studies have reported the interaction of HP35 to nanomaterials’ surface, we also compare our results with those obtained from other investigations. Noticeably, we observe both behaviors of adsorbed HP35 on different nanomaterials, that is, strong structural perturbations associated with unfolding events as well as retention of its native structure. Interestingly, HP35 commonly unfolds on flat nanomaterials’ surface (i.e., without defects), e.g., pristine graphene, C_3_N, boron nitride and BC_3_^45,47,48,53,54^, because the flat flawless surface allows the fast lateral shift of HP35, by which the critical residues (e.g., aromatic residues) may be exposed on the nanomaterials’ surface with the subsequent HP35 denaturation. Our simulation results show that the unfolding of HP35 on fluorinated graphene follows this fast lateral shifting mechanism. In addition, the second unfolding mechanism is called “anchoring-shifting”. In detail, basic residues of HP35 are tightly anchored by the negatively charged defects on the 2D nanomaterial, and simultaneously the other part of HP35 moves on the flat region around the defect. When these two events occur at the same time, the HP35 moves towards the loss of its tertiary structure. However, this mechanism should happened when there is a relatively large flat surface around the defect. The HP35 unfolding will not occur if the defects of the 2D nanomaterials are relatively close to each other, e.g., in phosphorene^49^, C_2_N^50^, C_3_N_4_^51^, C_3_N_3_^52^ and in α-phase phosphorene carbide^55^. The defects on these nanomaterials usually contain local charges, attracting the residues of HP35 with opposite charges, by which the HP35 is fully fixed on one position and the lateral movement of the protein on 2D nanomaterials’ surface is restrained, thus the HP35 protein structure remains intact. The inherent defects on some nanomaterials (e.g., phosphorene and α-phase phosphorene carbide) can weaken the interactions between the 2D nanostructure and HP35 resulting in the decreased possibility of exposing the critical residues, even though the HP35 can still rapidly shift on the surface of these nanomaterials. Combined, the 2D nanomaterials’ surface detailed features show a significant role to the HP35 protein adsorption mechanism. Finally, in this work, our purpose is to find if HP35 could unfold on fluorinated graphene. Along these lines, we could estimate the toxicity of the fluorinated graphene nanomaterial through the structural unfolding of this prototypical protein. Thus, the multiple folding pathways proposed for HP35 do not affect the conclusions reached here, even though the HP35 might have multiple debated folding pathways.

## Conclusion

In summary, we employ extensive MD simulation to investigate the impact of fluorinated graphene on the HP35 protein. Three simulation systems, each comprising three parallel simulations, are constructed. The MD results demonstrate that fluorinated graphene can induce the unfolding of the HP35 helical structure in certain cases, ultimately causing HP35 to lie on the fluorinated graphene surface. Subsequent analysis reveals that hydrophobic and van der Waals interactions predominantly govern the adsorption of HP35 to the fluorinated graphene surface, with electrostatic interactions playing a minor role. Additionally, we observe the complete loss of the hydrophobic core formed by three aromatic (phenylalanine) residues, crucial for maintaining the tertiary structure of HP35. Taking together, these perturbations to the native state suggest a significant structural damage induced by the physical adsorption of HP35 onto fluorinated graphene. Furthermore, the unrestrained and rapid movement of HP35 on fluorinated graphene leads to the exposure of the hydrophobic core, a critical mechanism facilitating the unfolding of HP35. Our MD simulations unveil the potential toxicity of fluorinated graphene and elucidate its underlying mechanism, providing valuable insights for the future application of this attractive nanomaterial.

### Supplementary Information

Supplementary Information.

## Data Availability

The datasets used and/or analysed during the current study available from the corresponding author on reasonable request.
