# Supplementary material for: Molecular dynamics simulations suggest the potential toxicity of fluorinated graphene to HP35 protein via unfolding the α-helix structure
Source: Sci Rep. 2024 Apr 22;14:9168. doi: 10.1038/s41598-024-59780-3 (PMC11035638; doi:10.1038/s41598-024-59780-3)
Supplement: Supplementary file 1 — Supplementary Information. [file 41598_2024_59780_MOESM1_ESM.docx]

**Simulation analysis method.**

The simulation data and analyses were conducted using GROMACS, VMD as well as the script. In detail, the structural conformations (including C3N, HP35 helices, and final binding conformations) were drawn by VMD software package. The contact number between HP35 and fluorinated graphene was calculated by the command of “gmx mindist”, wherein the atoms of protein having minimum distance to fluorinated graphene less than 0.5 nm were taken into account. Interaction energies were calculated via three processes, (1) the command “gmx grompp” generating a tpr file that includes two targeted energy groups, (2) rerunning the trajectories using the obtained tpr file, and (3) the command “gmx energy” generating every interaction energy. Hydrogen bond number of protein was calculated via the command “gmx hbond”. Q values were calculated via comparing the native contact number within residues at each time among the trajectory against that of the crystal structure of HP35. Root-mean-square deviation (RMSD) was computed using the command “gmx rms” through comparing the heavy atoms (i.e., non-hydrogen atoms) of HP35 at each time against those at first frame. The contact probability of each residue to fluorinated graphene was calculated via the formula: $contact probability=\frac{N_{contact}}{N_{total}}$; wherein $N_{contact}$ denoted the frame number that the corresponding residue contacted fluorinated graphene (we defined the contact residue with any heavy atom having a closest distance to any fluorinated graphene atom lower than 0.5 nm), and $N_{total}$ indicated the total frame number of the trajectory. Alignment of HP35 was plotted by VMD software via aligning the chosen HP35 structures (extracting every 500 ns from the trajectory and totally seven structures), wherein the HP35 colors were set by changing the “Occupancy” value from 0.1 to 0.7. The center of mass positions of HP35 were obtained using the command “gmx traj” in GROMACS. Mean square deviation (MSD) of HP35 moving along x-y plane was measured via the command “gmx msd”. Secondary structure of HP35 was measured with the command “do dssp” in GROMACS.


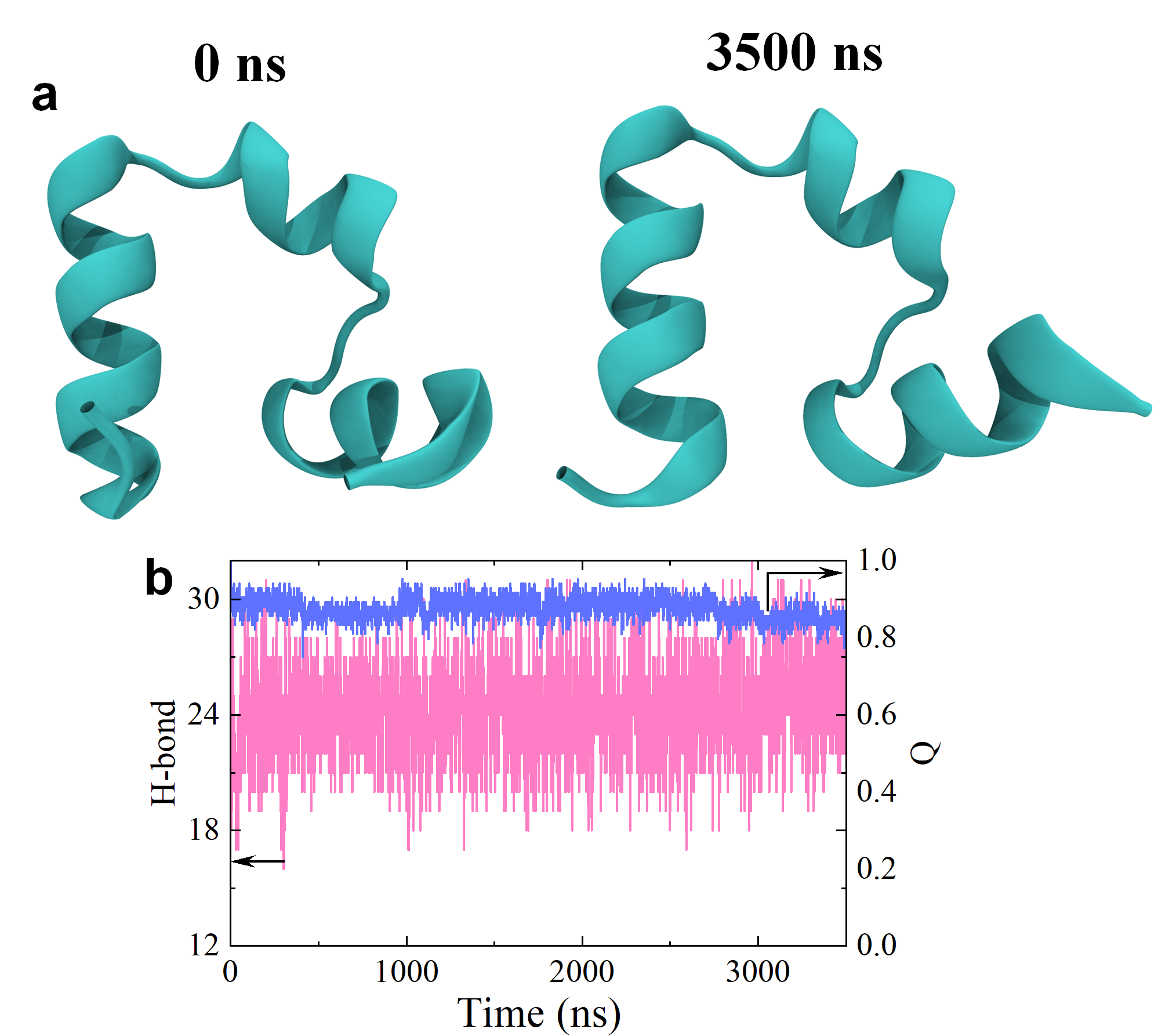


Figure S1. Simulation of an individual HP35 in solution. (a) Initial (0 ns) and final (3500 ns) conformations of HP35. (b) Hydrogen bond and Q value evolutions of HP35 in solution.


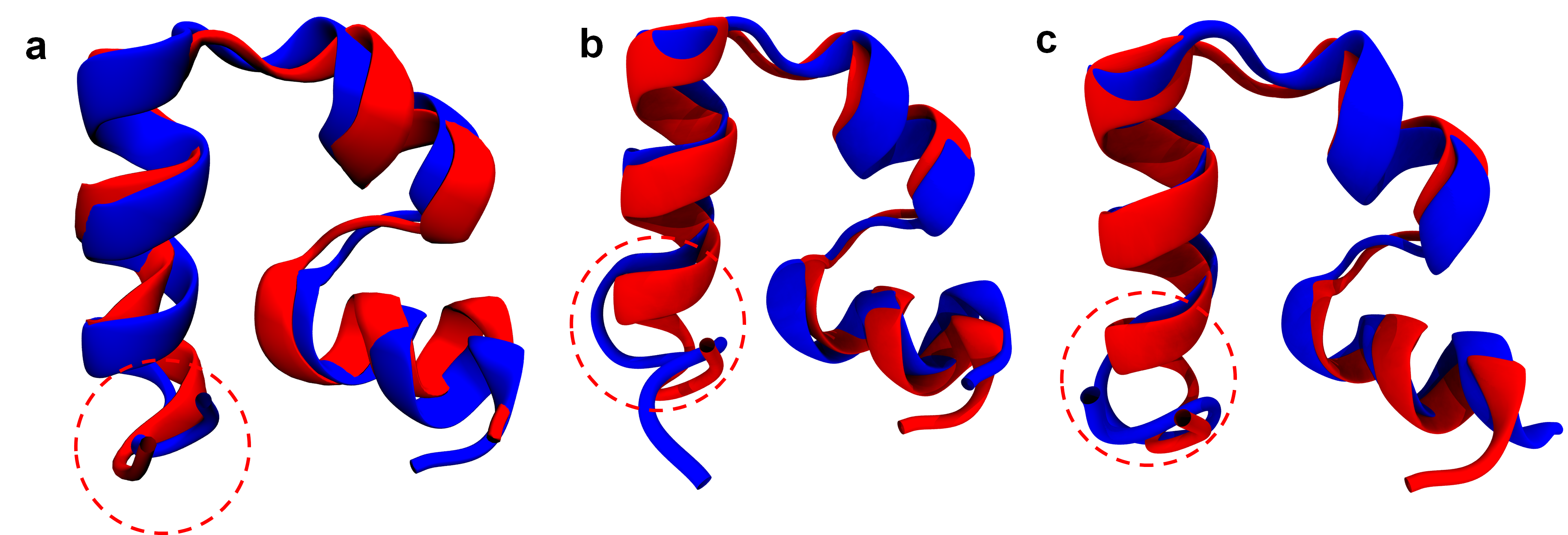


Figure S2. (a) Initial (red) and final (blue) conformations of HP35 in the simulation run-2 of sys-1. (b) Initial (red) and final (blue) conformations of HP35 in the simulation run-1 of sys-2. (c) Initial (red) and final (blue) conformations of HP35 in the simulation run-1 of sys-3. Red dashed circles denote the unfolding region.


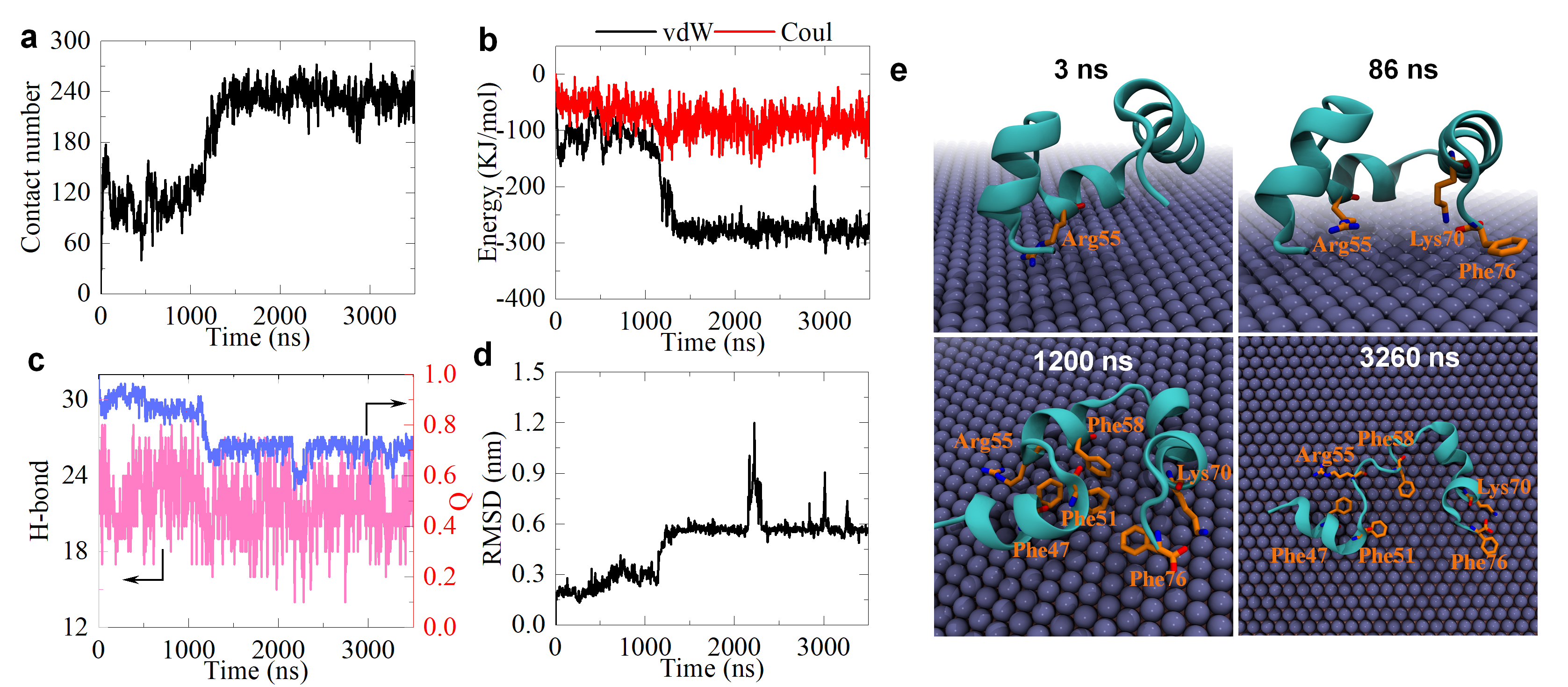


Figure S3. Analysis of the simulation run-2 of sys-2. (a) Atom contact number between HP35 and fluorinated graphene. (b) Interaction energies between HP35 and fluorinated graphene. vdW and Coul indicate van der Waals and Coulomb energies. (c) Hydrogen bond number and Q value of HP35. (d) Root mean square deviation (RMSD) of HP35. (e) Snapshots of HP35 binding to fluorinated graphene at some time points. The contacted basic and aromatic residues are highlighted.


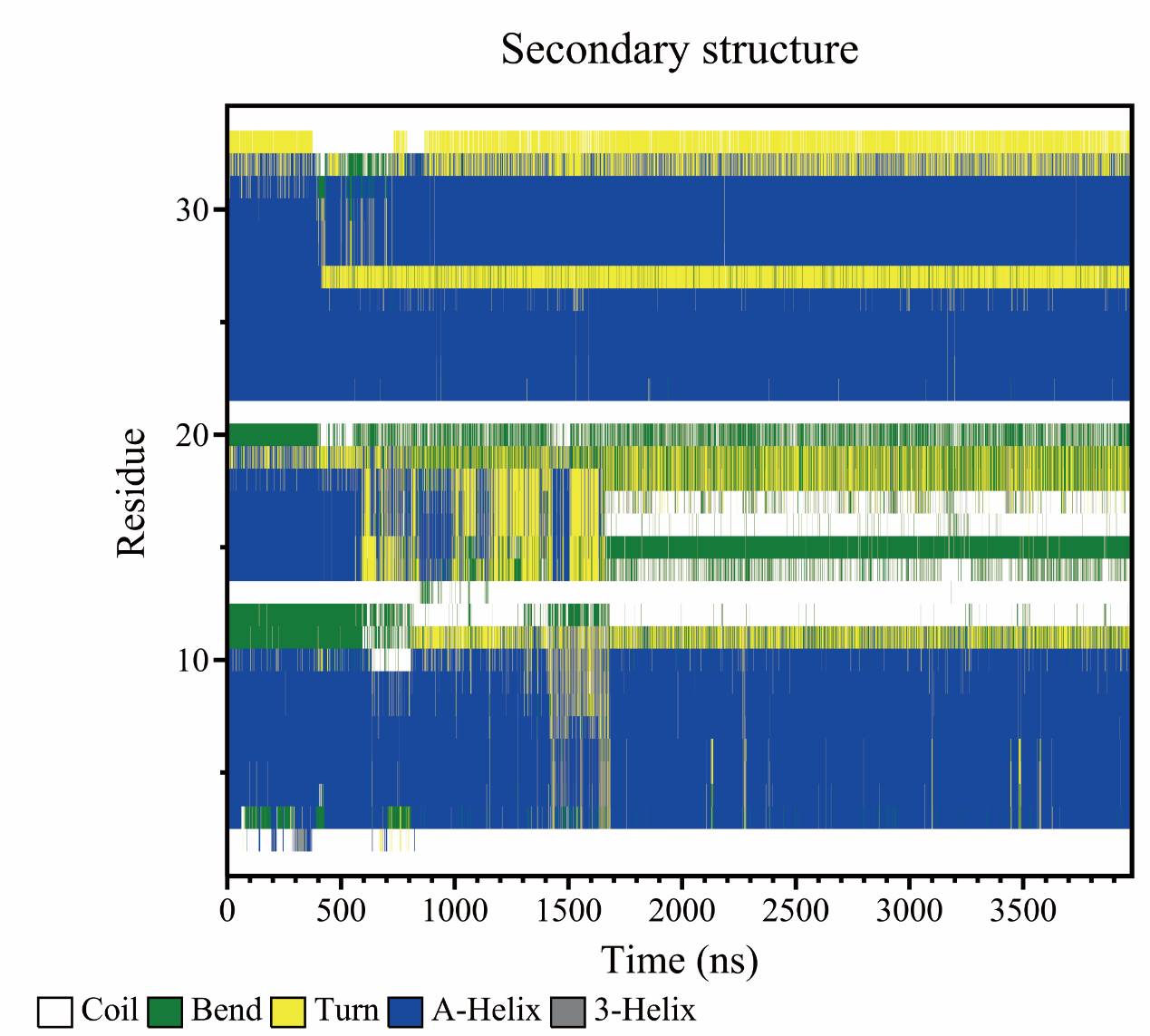


Figure S4. Secondary structure evolution of HP35 binding to fluorinated graphene. The data is obtained from the run-3 of sys-2.

Figure S5. Simply evaluated conformational entropy of HP35 as a function of simulation time. The data are obtained from the run-3 of sys-2.


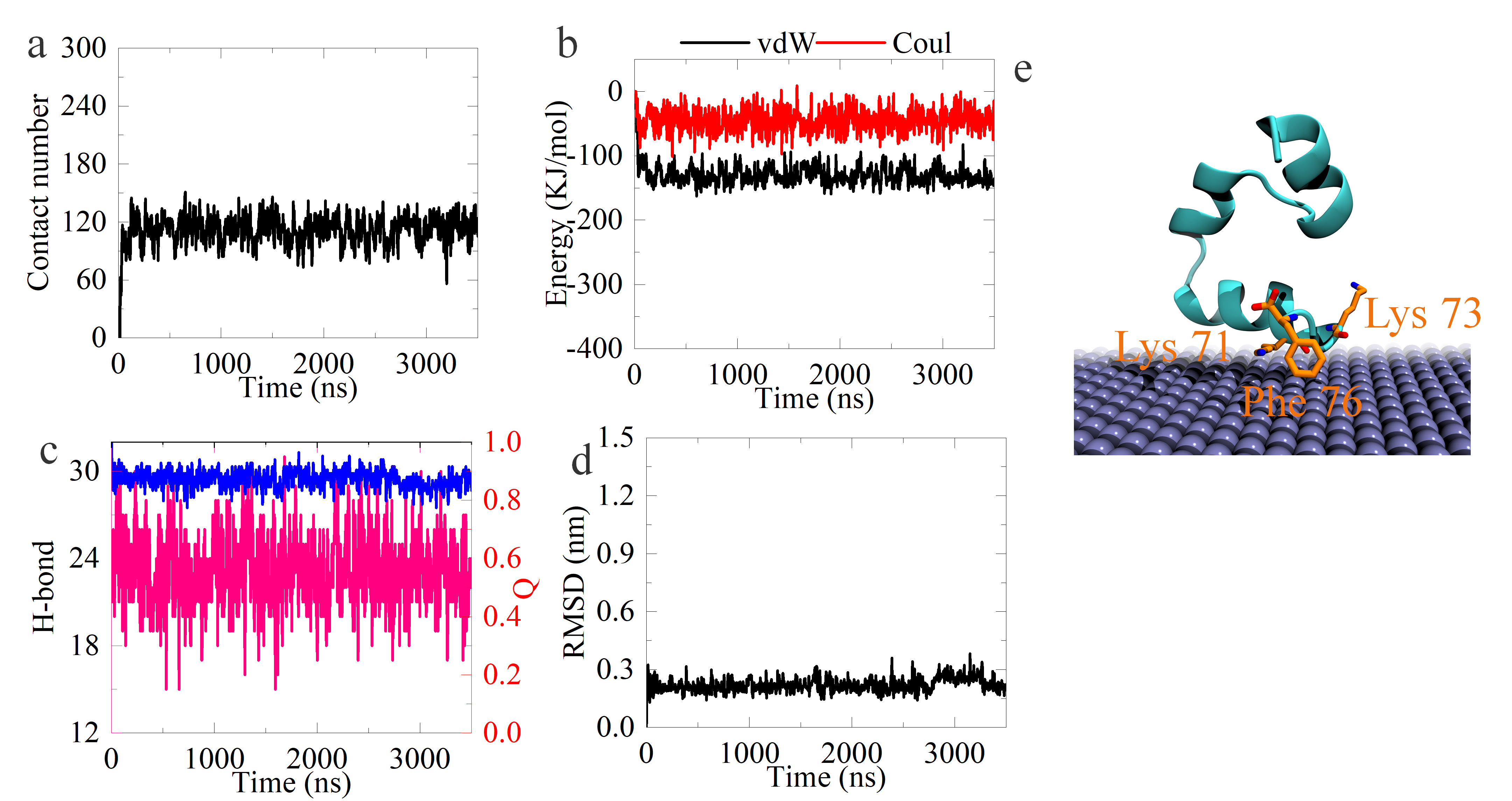


Figure S6. Analysis of the simulation run-1 of sys-1. (a) Atom contact number between HP35 and fluorinated graphene. (b) Interaction energies between HP35 and fluorinated graphene. vdW and Coul indicate van der Waals and Coulomb energies. (c) Hydrogen bond number and Q value of HP35. (d) Root mean square deviation (RMSD) of HP35. (e) Snapshot of HP35 binding to fluorinated graphene at the final frame of the trajectory. The contacted basic and aromatic residues are highlighted.


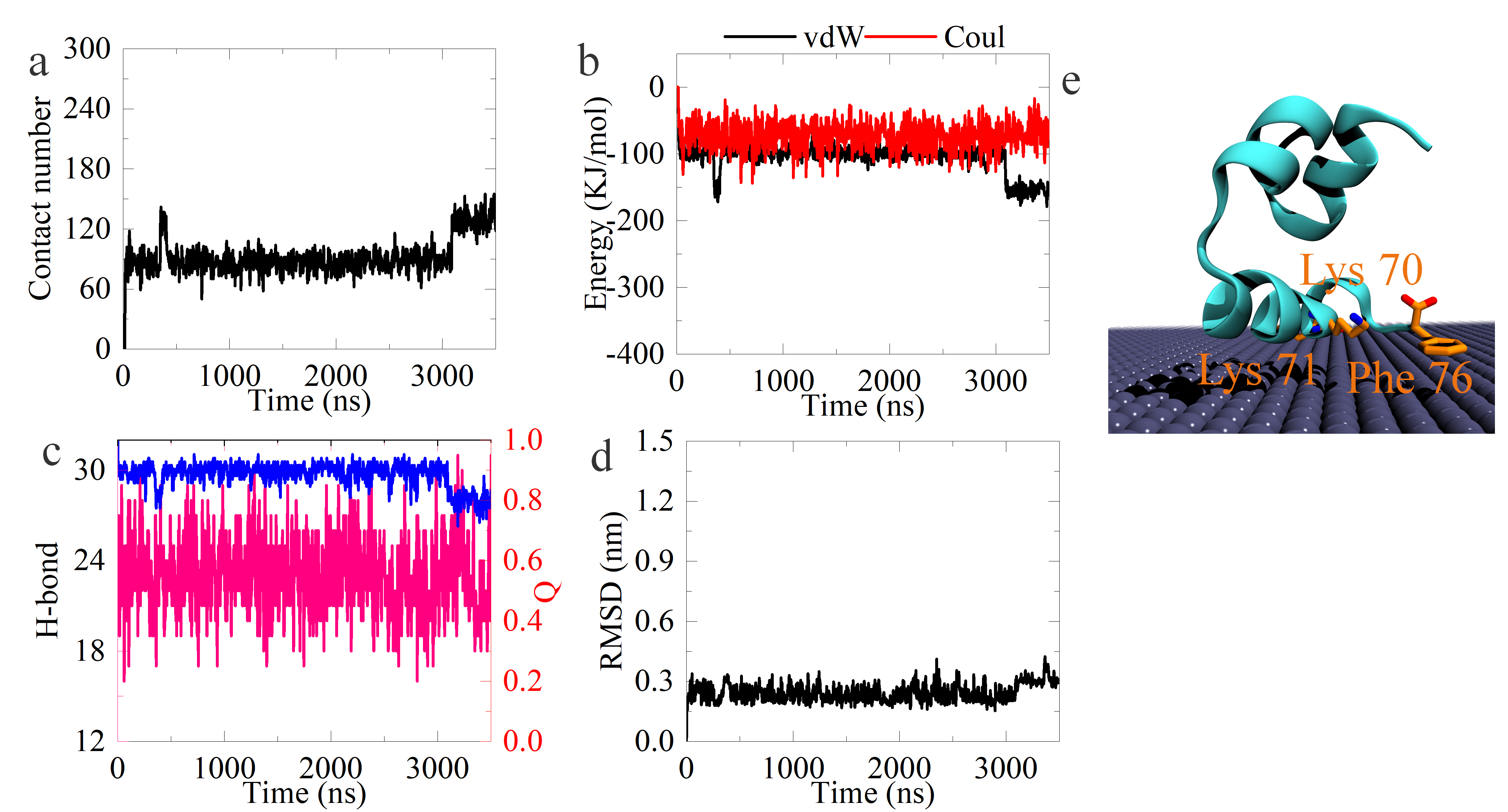


Figure S7. Analysis of the simulation run-2 of sys-1. (a) Atom contact number between HP35 and fluorinated graphene. (b) Interaction energies between HP35 and fluorinated graphene. vdW and Coul indicate van der Waals and Coulomb energies. (c) Hydrogen bond number and Q value of HP35. (d) Root mean square deviation (RMSD) of HP35. (e) Snapshot of HP35 binding to fluorinated graphene at the final frame of the trajectory. The contacted basic and aromatic residues are highlighted.


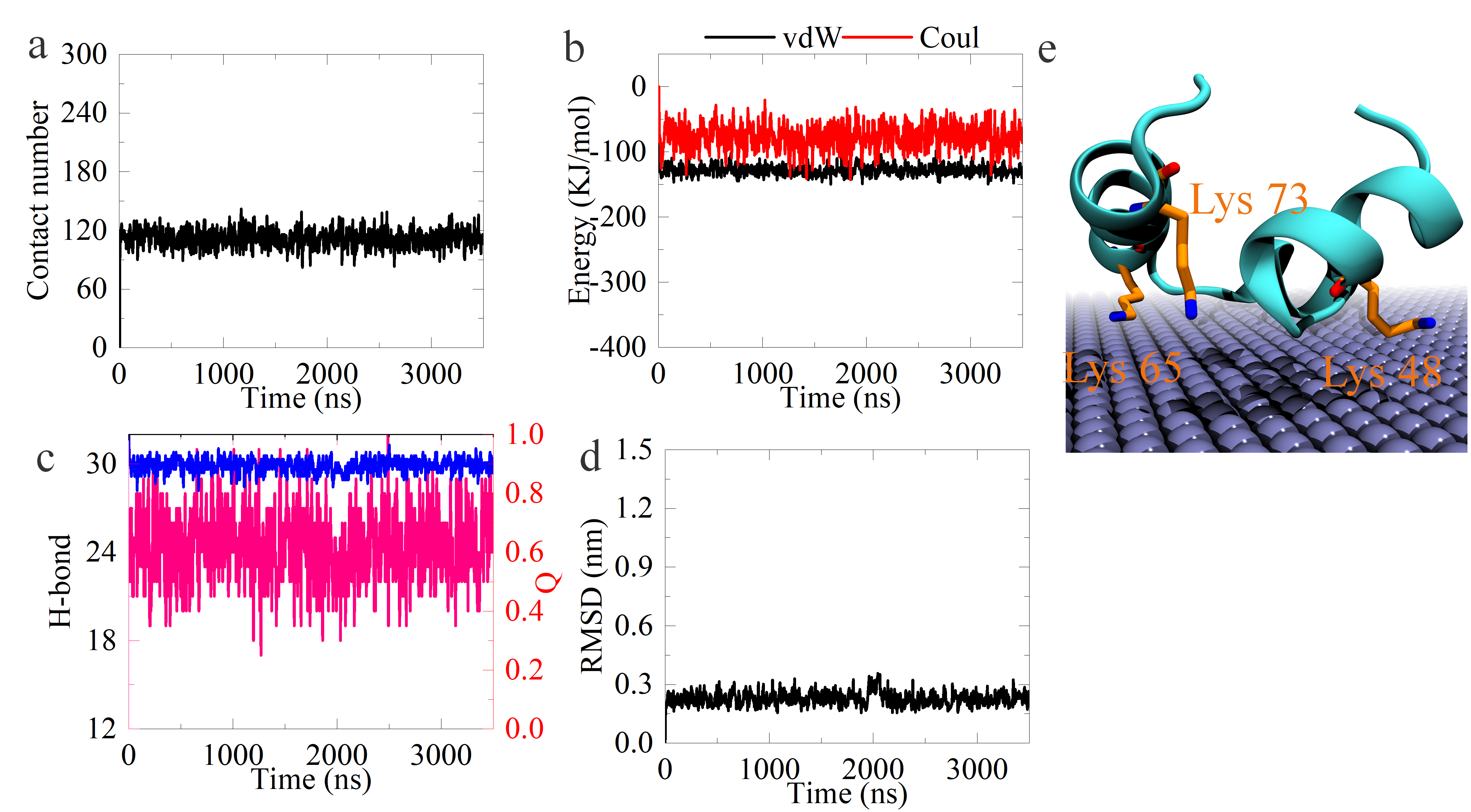


Figure S8. Analysis of the simulation run-3 of sys-1. (a) Atom contact number between HP35 and fluorinated graphene. (b) Interaction energies between HP35 and fluorinated graphene. vdW and Coul indicate van der Waals and Coulomb energies. (c) Hydrogen bond number and Q value of HP35. (d) Root mean square deviation (RMSD) of HP35. (e) Snapshot of HP35 binding to fluorinated graphene at the final frame of the trajectory. The contacted basic and aromatic residues are highlighted.


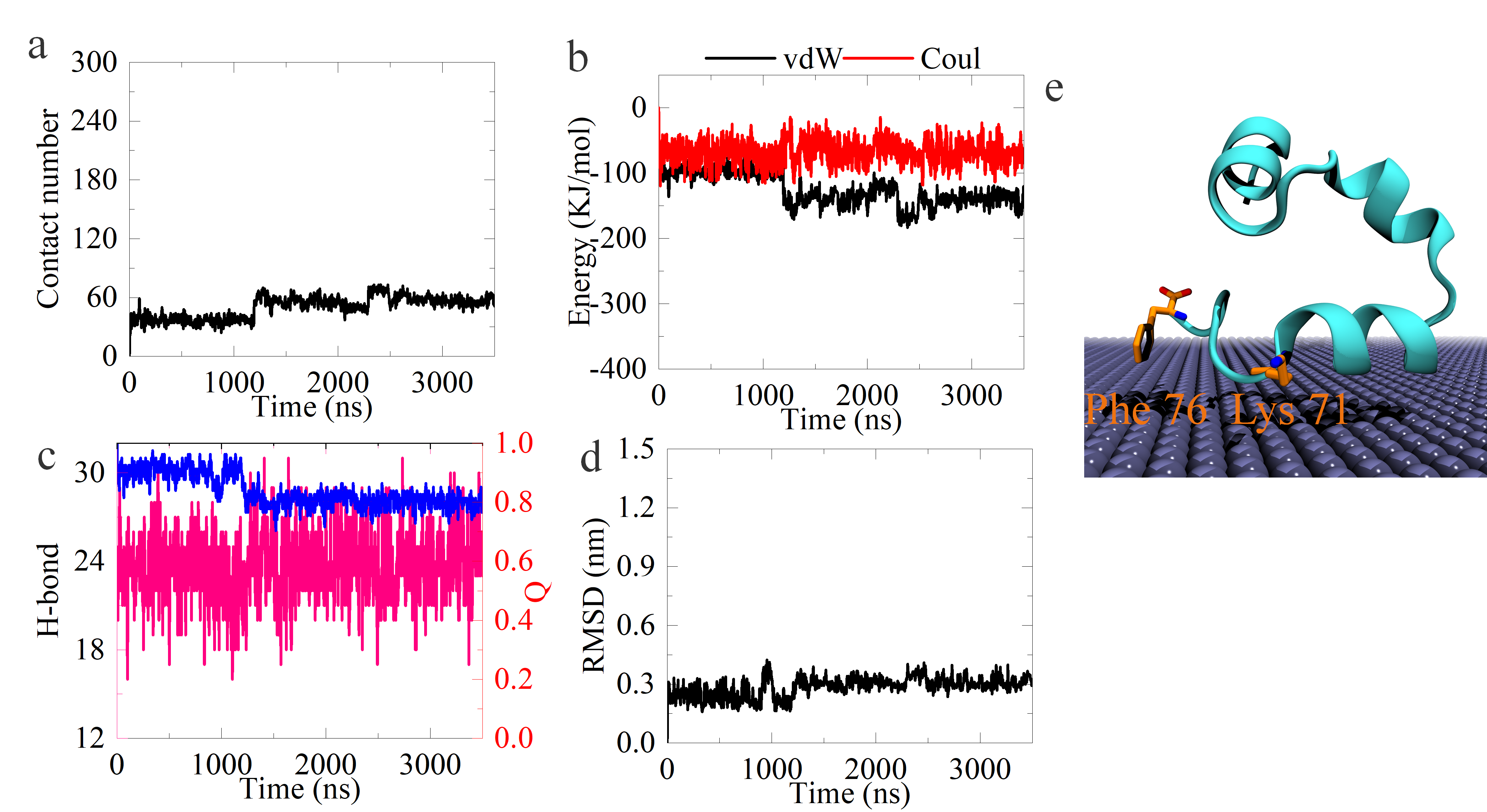


Figure S9. Analysis of the simulation run-1 of sys-2. (a) Atom contact number between HP35 and fluorinated graphene. (b) Interaction energies between HP35 and fluorinated graphene. vdW and Coul indicate van der Waals and Coulomb energies. (c) Hydrogen bond number and Q value of HP35. (d) Root mean square deviation (RMSD) of HP35. (e) Snapshot of HP35 binding to fluorinated graphene at the final frame of the trajectory. The contacted basic and aromatic residues are highlighted.


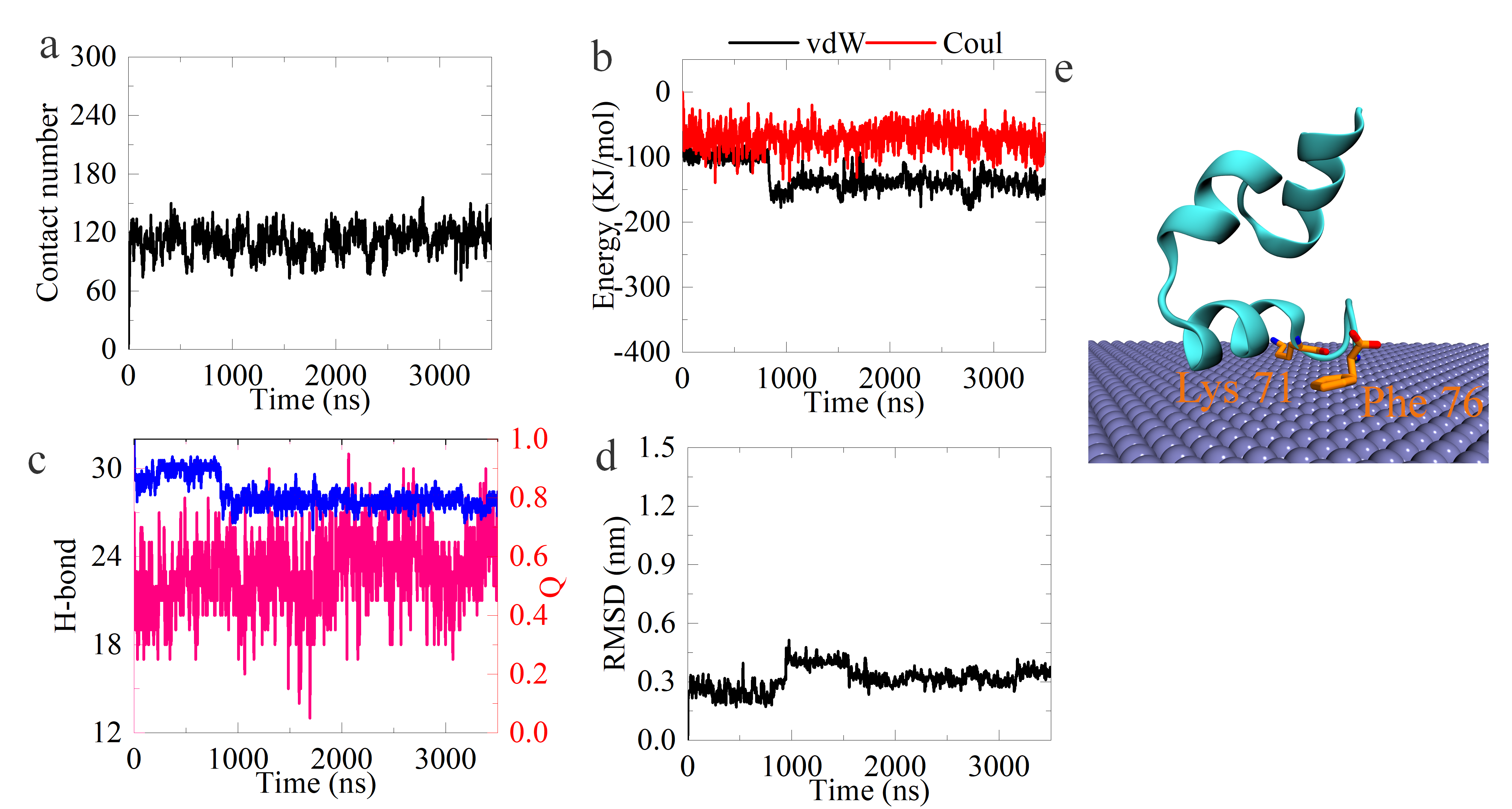


Figure S10. Analysis of the simulation run-1 of sys-3. (a) Atom contact number between HP35 and fluorinated graphene. (b) Interaction energies between HP35 and fluorinated graphene. vdW and Coul indicate van der Waals and Coulomb energies. (c) Hydrogen bond number and Q value of HP35. (d) Root mean square deviation (RMSD) of HP35. (e) Snapshot of HP35 binding to fluorinated graphene at the final frame of the trajectory. The contacted basic and aromatic residues are highlighted.


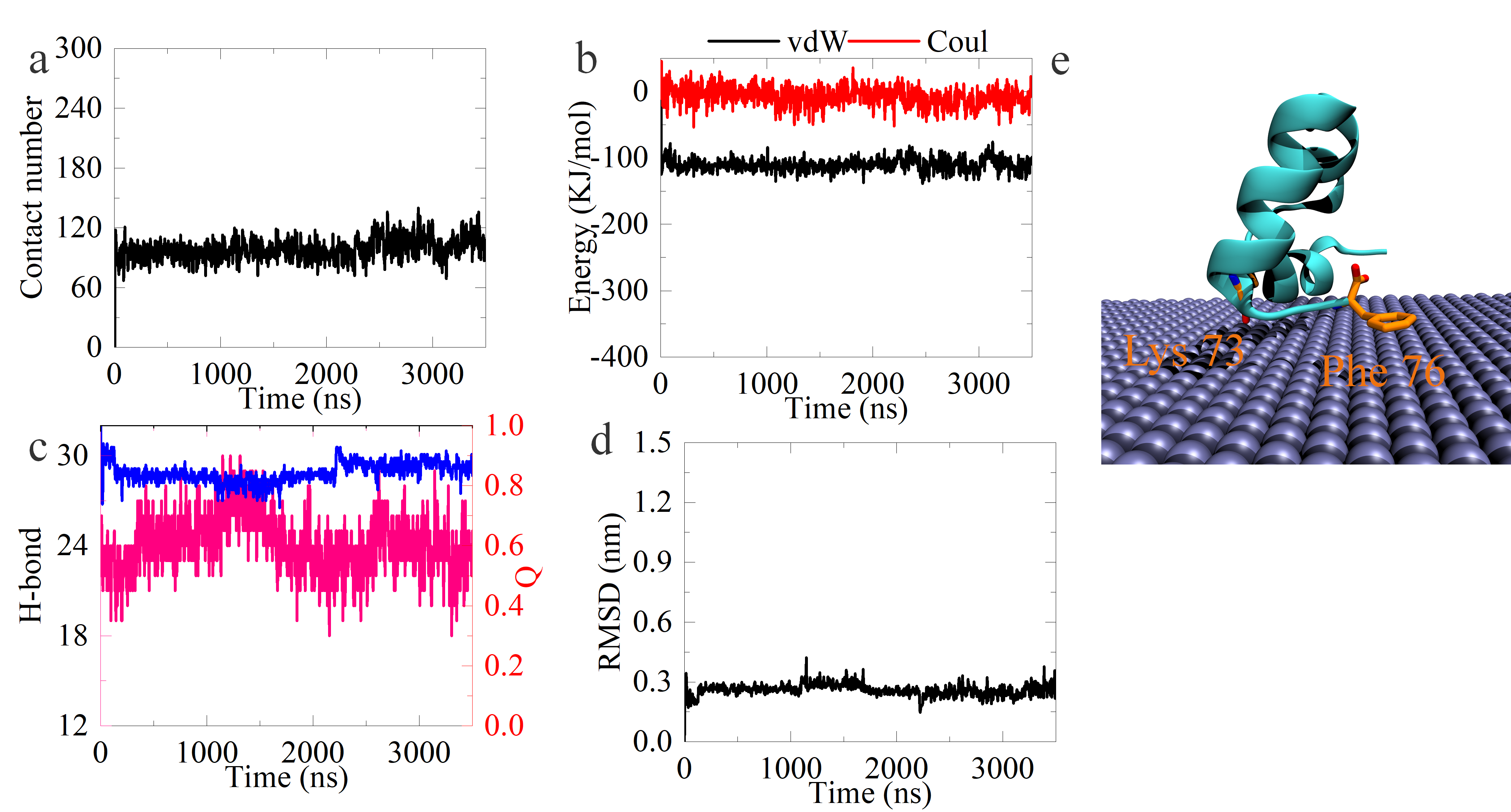


Figure S11. Analysis of the simulation run-2 of sys-3. (a) Atom contact number between HP35 and fluorinated graphene. (b) Interaction energies between HP35 and fluorinated graphene. vdW and Coul indicate van der Waals and Coulomb energies. (c) Hydrogen bond number and Q value of HP35. (d) Root mean square deviation (RMSD) of HP35. (e) Snapshot of HP35 binding to fluorinated graphene at the final frame of the trajectory. The contacted basic and aromatic residues are highlighted.


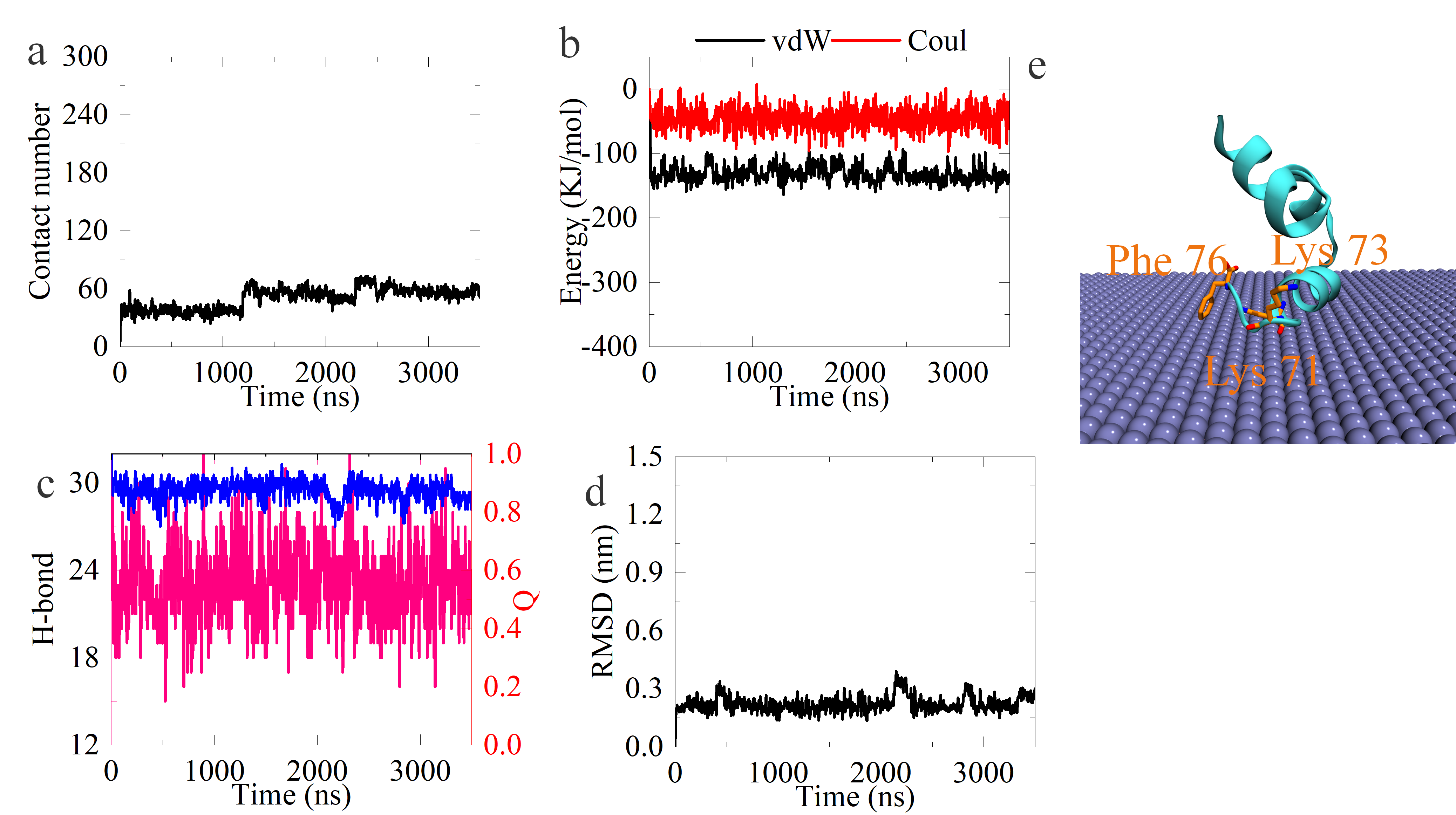


Figure S12. Analysis of the simulation run-3 of sys-3. (a) Atom contact number between HP35 and fluorinated graphene. (b) Interaction energies between HP35 and fluorinated graphene. vdW and Coul indicate van der Waals and Coulomb energies. (c) Hydrogen bond number and Q value of HP35. (d) Root mean square deviation (RMSD) of HP35. (e) Snapshot of HP35 binding to fluorinated graphene at the final frame of the trajectory. The contacted basic and aromatic residues are highlighted.

Table S1. Simulation number details.

| system name | HP35 protein only | sys-1 (HP35 rotating at 0˚) | sys-2 (HP35 rotating at 180˚) | sys-3 (HP35 rotating at 270˚) |
| --- | --- | --- | --- | --- |
| parallel simulation number | 1 | 3 | 3 | 3 |
